# Population Pharmacokinetics and Pharmacodynamics of Praziquantel in Ugandan Children with Intestinal Schistosomiasis: Higher Dosages Are Required for Maximal Efficacy

**DOI:** 10.1128/mBio.00227-16

**Published:** 2016-08-09

**Authors:** Amaya L. Bustinduy, David Waterhouse, Jose C. de Sousa-Figueiredo, Stephen A. Roberts, Aaron Atuhaire, Govert J. Van Dam, Paul L. A. M. Corstjens, Janet T. Scott, Michelle C. Stanton, Narcis B. Kabatereine, Stephen Ward, William W. Hope, J. Russell Stothard

**Affiliations:** a Department of Parasitology, Liverpool School of Tropical Medicine, Liverpool, United Kingdom; b Department of Clinical Research, London School of Hygiene and Tropical Medicine, London, United Kingdom; c Centro de Investigação em Saúde de Angola, Hospital Provincial, Caxito, Bengo, Angola; d Department of Life Sciences, Natural History Museum, Wolfson Wellcome Biomedical Laboratories, London, United Kingdom; e Center for Biostatistics, Manchester Academic Health Sciences Centre (MAHSC), University of Manchester, Manchester, United Kingdom; f Vector Control Division, Ministry of Health, Kampala, Uganda; g Department of Parasitology, Leiden University Medical Center, Leiden, The Netherlands; h Department of Molecular Cell Biology, Leiden University Medical Center, Leiden, The Netherlands; i Department of Molecular and Clinical Pharmacology, University of Liverpool, Liverpool, United Kingdom

## Abstract

Each year, millions of African children receive praziquantel (PZQ) by mass drug administration (MDA) to treat schistosomiasis at a standard single dose of 40 mg/kg of body weight, a direct extrapolation from studies of adults. A higher dose of 60 mg/kg is also acceptable for refractory cases. We conducted the first PZQ pharmacokinetic (PK) and pharmacodynamic (PD) study in young children comparing dosing. Sixty Ugandan children aged 3 to 8 years old with egg patent *Schistosoma mansoni* received PZQ at either 40 mg/kg or 60 mg/kg. PK parameters of PZQ racemate and enantiomers (*R* and *S*) were quantified. PD outcomes were assessed by standard fecal egg counts and novel schistosome-specific serum (circulating anodic antigen [CAA]) and urine (circulating cathodic antigen [CCA]) antigen assays. Population PK and PD analyses were performed to estimate drug exposure in individual children, and the relationship between drug exposure and parasitological cure was estimated using logistic regression. Monte Carlo simulations were performed to identify better, future dosing regimens. There was marked PK variability between children, but the area under the concentration-time curve (AUC) of PZQ was strongly predictive of the parasitological cure rate (CR). Although no child achieved antigenic cure, which is suggestive of an important residual adult worm burden, higher AUC was associated with greater CAA antigenic decline at 24 days. To optimize the performance of PZQ, analysis of our simulations suggest that higher doses (>60 mg/kg) are needed, particularly in smaller children.

## IMPORTANCE

Schistosomiasis is a neglected tropical disease, typically associated with chronic morbidity, and its control is a global health priority. Praziquantel (PZQ) is the only available antiparasitic drug and is often given out, as a single oral dose (40 mg/kg), to school-aged children by mass drug administration (MDA) schemes operating within preventive chemotherapy campaigns as endorsed by the World Health Organization (WHO). This current strategy has several limitations. (i) It excludes preschool children who can be patently infected. (ii) It delivers PZQ at a dose directly extrapolated from adult pharmacological studies. To address these problems, we conducted the first pharmacokinetic and pharmacodynamic study of young children within an area of Uganda where *Schistosoma mansoni* is hyperendemic. Our results demonstrate that a higher dose (>60 mg/kg) is required, especially in smaller children, and draw attention to the need for further optimization of PZQ treatment based on schistosome antigenic assays, which are more sensitive to pharmacodynamic markers.

## INTRODUCTION

Schistosomiasis is an important waterborne parasitic disease. An estimated 779 million people are at risk, and more than 50% of those are children (1, 2). Adult female schistosomes shed copious numbers of eggs each day that are either eliminated in host excreta or become lodged within host tissues. Chronic schistosomiasis is caused by the gradual accumulation of fibrotic lesions that occur around trapped eggs (2). To avert present and future morbidity, preventive chemotherapy campaigns, as endorsed by the World Health Organization (WHO), offer praziquantel (PZQ) via mass drug administration programs (MDA) to school-aged children.

PZQ is a pyrazinoisoquinoline drug licensed for oral use in children aged 4 years and above (3) and the only available treatment for the three principal *Schistosoma* species that cause human disease, *Schistosoma haematobium*, *S. mansoni*, and *S. japonicum*. The drug’s biovailability is enhanced by concomitant food administration (4), and it undergoes an extensive first-pass metabolism in the liver by the cytochrome P450 (CYP) system (CYP1A2, CYP3A4, CYP2BI, CYP3AD, and CYPC19) (5). PZQ is usually administered as a single dose between 40 and 60 mg/kg of body weight (6). PZQ damages the adult worm tegument by disrupting calcium ion channels—such damage may facilitate subsequent immunological attack and later clearance of the parasite. However, immature worms (schistosomulum) are insensitive to PZQ (1, 7). As adult worm death cannot be measured directly because of their intravascular location, the pharmacodynamics of PZQ is currently estimated using fecal egg clearance 3 to 4 weeks after treatment (8). Alternative biomarkers with potential prognostic value are measured by newly developed schistosome antigen assays using sera or urine (9, 10). Globally, the performance of PZQ is considered satisfactory, but there are some well-known exceptions that occur in some areas where schistosomiasis is hyperendemic. It is likely that these deficiencies are caused by insufficient drug exposure by inadequate initial dosing (11, 12).

Current formulations of PZQ are racemates consisting of two enantiomers [(−)*R* and (+)*S*], previously known as *levo* (l) and *dextro* (d) stereoisomers. A long-held belief is that the *R* isomer is significantly more active than the *S* form against *S. japonicum* and *S. mansoni*, although rodent studies are somewhat contradictory (13–16). In addition, *R*-PZQ is much more palatable than *S*-PZQ, and a monoenantiomeric formulation, currently in development, could reduce the dose by half, thus decreasing the total size of administered tablets (5, 13–18).

Despite being administered to an estimated 25 million children every year (19, 20), there is only limited pharmacokinetic and pharmacodynamic (PK-PD) information on the enantioselective properties of PZQ. The currently recommended dose of 40 mg/kg is a direct extrapolation from adults (5). Several epidemiological studies have revealed persistent egg patent schistosomiasis in preschool-aged children and in infants (11, 21–23) with poor worm clearance when given a standard dose at 40 mg/kg (4, 20, 24, 25). While this evidence has led to a change in WHO guidelines that now promote access to PZQ in younger children (9, 21, 26), detailed PK-PD studies are still lacking. We report here on the first PZQ PK-PD study in children aged 3 to 8 years infected with *S. mansoni*.

## RESULTS

**FIG 1:**
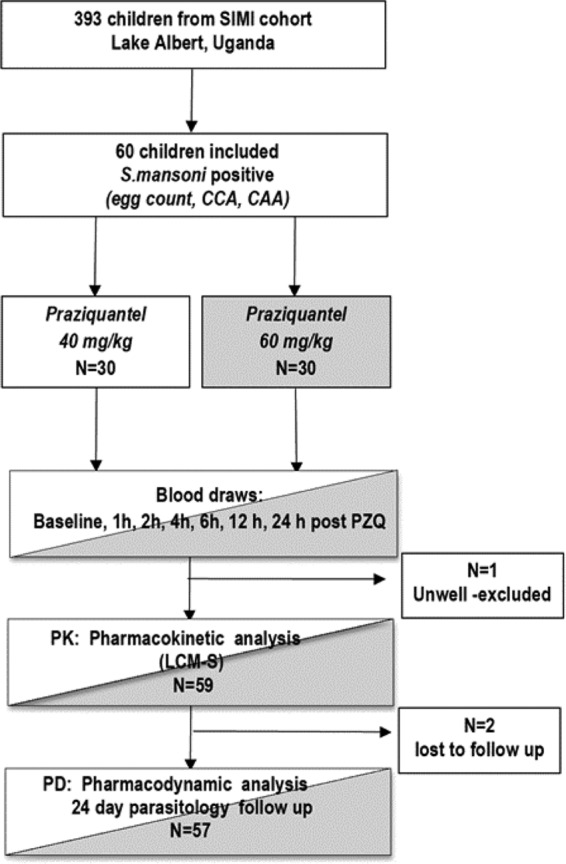
Design and basic methodology of the study. The SIMI cohort refers to the Schistosomiasis in Mothers and Infant Study.

A total of 60 children were enrolled in the study, and 59 completed the study protocol. One child was excluded 6 h into the study because of an acute febrile illness that was later diagnosed and treated as falciparum malaria (Fig. 1). No child tested positive for HIV (Table 1). Overall, there were no major clinical adverse events that were identified. All unpleasant symptoms, mostly sweating, abdominal pain and cramps, nausea, vomiting, headache, dizziness, and sleepiness, peaked between 1 and 4 h after receipt of PZQ and subsided between 1 and 3 h after the onset of symptoms. The side effects observed by the study pediatrician (ALB) were greater than the self-reported ones. The most common ones were sweating, abdominal pain and cramps, sleepiness, and headache. These symptoms were significantly greater in the 60-mg/kg arm (see Fig. S1 in the supplemental material). Fifty-eight children were available at the 24-day follow-up.

**TABLE 1:** Baseline characteristics of children enrolled in the study

Characteristic^a^	Total no. of children (*n* = 60)	No. of children or parameter value for children given the following praziquantel dose:		*P* value^b^
		40 mg/kg (*n* = 30)	60 mg/kg (*n* = 30)	
Demography				
Bugoigo village	23	11	12	>0.99
Walukuba village	37	19	18	
Age, yr [mean (range)]	60	6.4 (3–8)	6.3 (3–8)	0.79
3- to 5-year-old (preschool)	17	7	10	0.49
6- to 8-year-old (school-aged)	43	23	20	
Females	38	19	19	>0.99
Males	22	11	11	
Wt, kg [mean (range)]	60	21.8 (15–30)	23.0 (15.1–34)	0.26
Ht, cm [mean (range)]	60	119.4 (101–138)	118.8 (101–139)	0.80

Hematology				
Hemoglobin [mean (95% CI)]	59	10.6 (7.3–14.7)	11.3 (10.8–11.8)	0.048
Anemia^c^	59	23	14	0.062

Parasitic infection				
*S*. *mansoni* epg stool [arithmetic mean (CI)]	60	950.4 (324–1,576)	491.0 (249–733)	0.19
Heavy (>400 epg)	23	12	11	1.0
Medium (100–399 epg)	17	8	9	
Light (1–100 epg)	19	9	10	0.015
No eggs (CCA-positive)	1	1	0	
CCA in urine				
+	9	5	4	0.785
++	13	6	7	0.613
+++	33	16	17	>0.99
NA	5	3	2	
CAA concn in plasma, pg/ml [mean (95% CI)]	55	56746 (100–100,839)	43,217 (224–185,672)	0.39
Malaria SD RDT				
+	32	16	16	*P* > 0.99
++	13	7	6	
Negative	14	7	7	
NA	1	0	1	

a Abbreviations: CCA, circulating cathodic antigen; CAA, circulating anodic antigen; 95% CI, 95% confidence interval; NA, not available; RDT, rapid diagnostic text.

b *P* value is the difference between groups by Fisher exact test or Student’s *t* test.

c Anemia defined here as hemoglobin level of <11.5 g/dl.

### PZQ pharmacokinetics.

**FIG 2:**
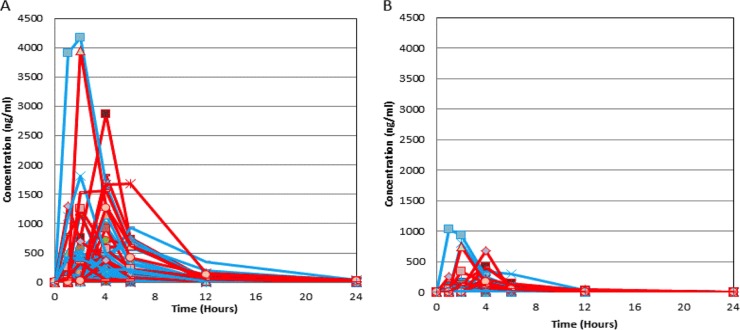
Individual PZQ levels by dosing arm. (A) *S*-PZQ and (B) R-PZQ. The 40-mg/kg dosing arm is indicated in blue, and the 60-mg/kg dosing arm is indicated in red.

**FIG 3:**
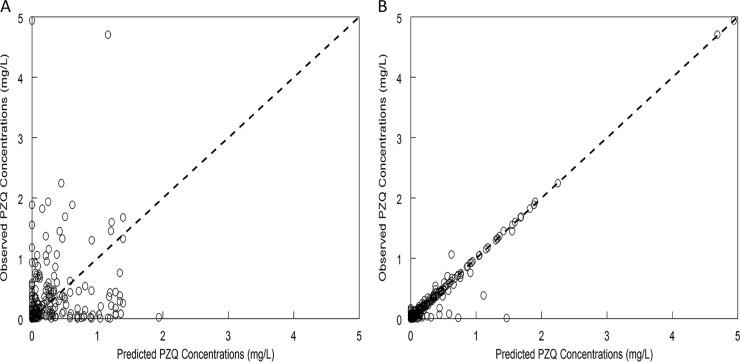
Observed versus predicted values before (A) and after (B) the Bayesian step.

The individual plasma PZQ enantiomer concentration-time profiles are shown in Fig. 2. There was marked pharmacokinetic variability in the concentrations of both the *R* and *S* isoforms. Table 2 shows pharmacokinetic parameter values at different doses for each enantiomer. This was evident in both the “spaghetti plot” and in the coefficient of variation (CV) of the population PK estimates for clearance and volume, which was >60% from both population PK models (Table 3). There was no relationship between any of the Bayesian estimates of the parameter values and any of the available covariates. Thus, the standard base model was used. The fit of the model to the data was satisfactory, although there was a degree of bias when the population parameter values were used. The observed-predicted plots both before and after the Bayesian step are shown in Fig. 3

**TABLE 2:** Pharmacokinetic parameter values derived from the 60 children receiving two different doses of praziquantel^a^

Dose and PZF enantiomer	*C*_max_ (µg/ml)	*T*_max_ (h)	AUC_0-∞_ (µg ⋅ h/ml)	*t*_1/2_ (h)	*T*_lag_
40 mg/kg					
*S*-PZQ	581.3 (677)	3.33 (2.7–3.9)	2.62 (2.8)	2.96 (2.2)	0.07 (0.2)
*R*-PZQ	131.1 (238)	3.28 (2.6–3.9)	0.50 (0.59)	3.48 (2.3)	0.46 (0.7)
60 mg/kg					
*S*-PZQ	695.56 (810)	3.23 (2.6–3.8)	2.58 (2.5)	3.02 (1.7)	0.13 (0.35)
*R*-PZQ	144.95 (185)	3.13 (2.6–3.6)	0.43 (0.23)	4.36 (4.7)	0.72 (0.88)

a Values are means, with standard deviations shown in parentheses. The values in parentheses for *T*_max_ show range. Abbreviations: *C*_max_, maximum concentration of drug in serum; *T*_max_, time to maximum concentration of drug in serum; *t*_1/2_, half-life; *T*_lag_, lag time (time delay between drug administration and first observed concentration above the lower limit of quantification in plasma).

**TABLE 3:** Population pharmacokinetic parameter values derived from the 59 children receiving praziquantel^a^

Pharmacokinetic parameter	Mean	Median	SD	CV%
*K_a_* (h^−1^)	14.89	9.88	13.31	89.36
SCL/*F* (liter/h)	608.02	677.59	320.10	52.65
*V*/*F* (liter)	473.97	503.90	244.80	51.65
*K*_cp_ (h^−1^)	25.90	22.28	18.89	72.92
*K*_pc_ (h^−1^)	33.30	25.71	26.08	78.32
*T*_lag_ (h)	1.94	1.67	1.13	58.34

a Abbreviations: *K_a_*, first-order rate constant connecting the gut with the bloodstream; SCL, clearance of PZQ; *K*_cp_ and *K*_pc_, first-order compartmental transfer rate constants connecting the central and peripheral compartments; *V*, volume of the central compartment; *F*, oral bioavailability of PZQ, which was not estimated in this study; CV%, coefficient of variation as a percentage.

### PZQ pharmacodynamics. (i) *S. mansoni* cure rate and egg rate reduction by fecal examination.

Both overall cure rate (CR) and egg reduction rate (ERR) were used to assess the parasitological response to PZQ. There were no statistically significant differences in the day 24 stool egg detection CR or ERR between the two doses of PZQ (Table 4). The odds ratios from logistic regression models of the cure rate are shown in Table 5 There is some evidence for an association of cure rate with gender, weight, and initial parasite load, but these would not be considered statistically significant after allowing for the number of parameters tested. As noted above, there is no significant association between cure and administered PZQ dose. In contrast, total PZQ AUC_0–24_ (area under the concentration-time curve from 0 to 24 h) and in particular the S form are associated with increased cure rates. The Akaike information criterion (AIC) measures of goodness-of-fit provide some positive evidence in favor of the *S* form, but there was no strong evidence than it necessarily has better explanatory value than total AUC. Furthermore, it should be noted that the *R* form was present in much lower concentrations, and it is possible that drug exposure (in terms of AUC) was not be accurately quantified. There were insufficient data (with only 14 “noncured” events) to allow exploration of multivariable models.

**TABLE 4:** Egg reduction rate and cure rate for *S. mansoni* egg output at 24-day follow-up^a^

PZQ dose or *P* value	ERR (%) [95% CI]	CR (%) [95% CI]
40 mg/kg (*n* = 30)	82 [70–90]	70 [52–83]
60 mg/kg (*n* = 28)	91 [74–98]	82 [64–92]
*P* value	0.24^b^	0.36^c^

a Abbreviations: ERR, egg reduction rate; CR, cure rate; 95% CI, 95% confidence interval.

b 95% CI and difference in rates computed using quasibinomial model.

c Exact binomial 95% CI calculated by Fisher’s exact test.

**TABLE 5:** Effects of disease burden, sex, weight, and drug on cure rate^a^

Factor or parameter	Unadjusted OR^b^	*P* value	Adjusted OR^c^	*P* value	AIC^d^
Baseline *S*. *mansoni*intensity (by eggs per gram)	0.99 (0.98–1.00)	0.047	0.99 (0.98–1.00)	0.047	
Females	3.18 (0.89–11.3)	0.068	3.65 (0.94–14.2)	0.056	
Wt (per kg)	1.28 (1.04–1.57)	0.016	1.27 (1.03–1.57)	0.022	
PZQ dose (per mg/kg)	1.03 (0.97–1.10)	0.29	1.02 (0.96–1.09)	0.48	64.8
*R*-PZQ AUC (log)	1.67 (0.85–3.30)	0.13	1.57 (0.77–3.20)	0.20	63.6
*S*-PZQ AUC (log)	2.98 (1.40–6.32)	0.004	2.99 (1.36–6.59)	0.005	**54.1**
Total PZQ AUC (log)	2.31 (1.21–4.40)	0.009	2.24 (1.15–4.37)	0.015	58.0

a Logistic regression unadjusted and adjusted for baseline S. mansoni severity of infection.

b OR, odds ratio. The 95% CI values are shown in parentheses.

c The adjusted odds ratio (OR) was adjusted for baseline severity only. The 95% CI values are shown in parentheses.

d The Akaike information criterion (AIC) values of the fitted models are shown for the various dose measures; lower values indicates a better fit, and differences of 2 to 6 would be regarded as positive evidence in favor of the selected dose measure. while >6 would constitute strong evidence in favor of that measure. The chosen model for simulations based on the best AIC is shown in boldface type.

### (ii) *S*. *mansoni* cure rate by plasma CAA levels and urine CCA.

**FIG 4:**
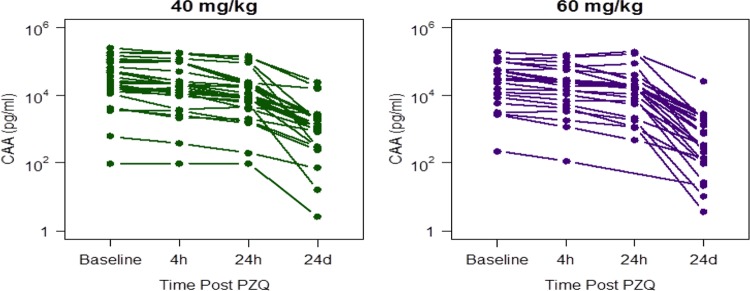
Individual CAA levels at baseline and 4 h, 6 h, 24 h, and 24 days post-PZQ for the two dose levels.

There was a sustained drop in CAA levels at 24 days. However, no children at 24 days follow-up achieved negative CAA levels (<10 pg/ml) (Fig. 4). Children that had lower PZQ AUC_0–24_ levels at baseline had higher CAA levels at 24 days follow-up, although this did not reach statistical significance (*P* = 0.071). Urine CCA negativity was achieved in only 7/58 (12%) children at follow-up (2 in the 40-mg/kg arm and 5 in the 60-mg/kg arm).

**FIG 5:**
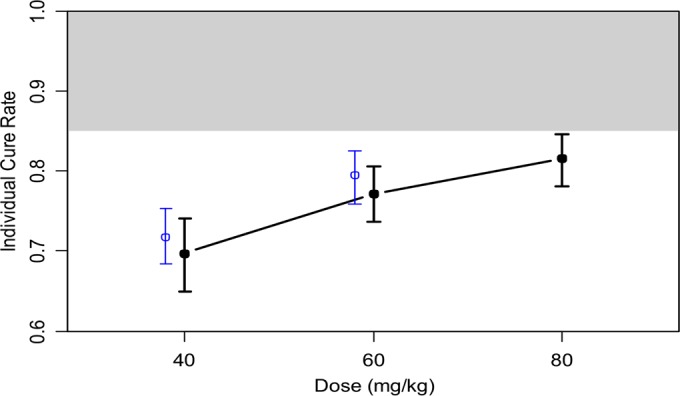
Model with individual cure rates as outcomes adjusted for infection intensity and PZQ AUC. The model estimated cure actual patient data (*n* = 60) for the doses given in the study (40 mg/kg and 60 mg/kg) are shown in blue. Data for simulated patients (*n* = 5,000) for three different doses (40 mg/kg, 60 mg/kg, and 80 mg/kg) are shown in black. Error bars on the simulation results represent the uncertainty (interquartile range) in the simulation due to parameter variability. The shaded area indicates the WHO target of a >85% cure rate.

Monte Carlo simulations were performed for three different dose regimens (40 mg/kg, 60 mg/kg, and 80 mg/kg). The results of the Monte Carlo simulation are shown in Fig. 5 with individual cure rates for the different PZQ doses in study patients compared to simulated patients, adjusting for baseline intensity of infection and AUC. Only 75% of patients receiving the highest PZQ dose of 80 mg/kg had an estimated cure rate of >85%. Whether dosing should be further stratified on the basis of other covariates, such as sex and weight, is possible but requires larger studies.

## DISCUSSION

Since 1984, PZQ has been the standard of care for all forms of schistosomiasis with a recent focus on WHO-approved preventive chemotherapy campaigns that target school-aged children. Despite this, relatively little is known about PZQ drug exposure-effect relationships which underpin therapeutic efficacy in children. Our PK-PD study is the first to quantify the drug exposure-response relationships using both traditional parasitological (fecal egg counts per gram of stool [epg]) and novel, more-sensitive schistosome antigenic assays (CCA and CAA). This is also the first study of *S. mansoni* in humans showing differences in enantiomeric activity on worm reduction. The probability of cure was strongly associated with high levels of *S*-PZQ. The *R*-PZQ form was eliminated much faster than the *S*-PZQ form, meaning that estimates of drug exposure may be less precise and consequently making it difficult to establish definitive drug exposure-response relationships. We postulate that there is still the possibility that *R*-PZQ could be biologically active, but its exposure and activity are better reflected by the more-abundant *S*-PZQ form.

*In vitro* and murine studies support the antischistosomal activity of *R*-PZQ in *S. japonicum* (5). However, there is still some controversy regarding the relative potency of PZQ enantiomers against *S. mansoni*. Two murine studies with *S. mansoni* have demonstrated that *R*-PZQ has limited activity when assessed by worm reduction and tegumental damage (32% worm reduction) (13, 14, 16, 17, 27). A recent study also found *R*-PZQ to be the active enantiomer in *S. mansoni*-infected mice but found no activity of its metabolites (15). Clinical correlation, however, is scant. A PD study in China of 278 patients with *S. japonicum* compared a single dose of *R*-PZQ (20 mg/kg) to racemic PZQ (40 mg/kg) with posttreatment cure rates of 94.8% and 97.1%, suggesting that *R*-PZQ is the active enantiomer (28). Due to such discrepancies between our study in *S. mansoni* human subjects and existing preclinical data, there is an urgent requirement to better understand species-specific enantiomeric activity of PZQ.

PZQ exhibits considerable interindividual pharmacokinetic variability. The plasma half-life is short (ca. 2 to 4 h), and there is almost no parental drug detected in serum 12 to 24 h after dosing. The majority of PK variability is unexplained. An allometric relationship where weight affects clearance via a 0.75 scaling exponent may well be the most appropriate scaling function but would require PK data from children with a wider range of weights to investigate this further. Other sources of variation in absorption likely result from food effects and underlying intestinal disease, as well as pharmacogenetic determinants of drug clearance. PZQ is metabolized by CPY3A4, CYP1A2, and CYP2C19, which are known to exhibit polymorphisms (29) and may be held accountable, as age-related CYP maturity may explain some of the pharmacokinetic variability (30). A potential impact of CYP polymorphisms in different age and weight groups warrants further scrutiny.

PZQ binds the tegument of the adult worm, and host-immune effectors are ultimately responsible for elimination of the parasite (31). Despite this, there is persistent antischistosomal activity, as evidenced by a progressive decline in excreted eggs in stool, and in circulating antigen levels (CAA) in plasma that extends well beyond the time that plasma drug concentrations become undetected (Fig. 4). This hysteresis (i.e., apparent disconnect between drug exposure and various measures of therapeutic effect) may have several explanations. First, it is possible that PZQ has a prolonged mean residence time within the schistosomal tegument or contiguous host tissues and is able to exert a persistent pharmacological effect long after serum drug concentrations are undetected. Second, it is possible that very short exposure to PZQ is sufficient to initiate changes in the tegument that enable immune effectors to kill the parasite. The distinction requires further preclinical studies.

Despite the fact that this study was not designed to detect differences in therapeutic outcomes with the two treatment regimens, we found a strong relationship between total drug AUC and parasitological cure while there was no detectable impact of administered dose. When CR (i.e., no eggs in stool at follow-up) was analyzed as a final-outcome measure, higher total PZQ AUCs were associated with increased cure. Several decades of antimicrobial PK-PD research have demonstrated that dose is a notoriously inaccurate measure of actual drug exposure in clinical settings, as exemplified by the between-patient variation (Fig. 2). The ability to resolve drug exposure-effect relationships in this study is almost exclusively a function of quantifying drug exposure in terms of AUC_0–24_ rather than the reported dose. The extreme PK variability is the likely explanation for the previous observation that better response rates are not observed with given higher dosages (32–34). Overall, higher PZQ dosages than those administered in this study are required to achieve acceptable cure rates (defined as a cure rate of >85%) (3). A target AUC should be set for future studies delivering higher doses than the ones used for this study.

One of the key strengths of our study was inclusion of novel, more-sensitive antigenic assays (plasma CAA and urine CCA) used alongside traditional parasitological methods. Although very poor cure rates were obtained when CCA was used as a measure of cure (12%), a higher proportion of children achieved assay negativity when given a 60-mg/kg dose (35). Similarly, plasma CAA levels experienced a high drop at follow-up but at 24 days after PZQ (mean, 2,582 pg/ml) were still above the thresholds for negativity (<10 pg/ml) (Fig. 4). However, when taken together, a single PZQ dose in this area where schistosomiasis is highly endemic failed to achieve antigenic cure, which is indicative of viable worms which may simply be due to temporary cessation of egg excretion by adult females. Facultative egg excretion is a well-known confounder of parasitological methods and highlights that future drug development programs should consider the use of these antigens (CCA and CAA) as better measures of drug efficacy, over and above fecal egg patency.

Even with the limitation of a small sample size to effectively detect PD changes, the results of our PK-PD analyses firmly suggest that 40 mg/kg is too low a dose. As a matter of clinical urgency, we suggest that the following studies are needed: (i) further PK-PD assessment(s) of higher doses in young children, (ii) development of an appropriate pharmacodynamic index linking drug exposure with antiparasitic effect(s), and (iii) clarification of *R*-PZQ and *S*-PZQ activity across all *Schistosoma* species infections before monoenantiomeric formulations are introduced.

## MATERIALS AND METHODS

### Ethics statement.

Ethical approval was obtained from the Liverpool School of Tropical Medicine (application no. 5538/2009) and the Uganda National Council for Science and Medical Research. Detailed information sheets were provided to parents and/or guardians. Informed written consent was obtained from the child’s guardian, and verbal assent was also obtained from children who were able to do so.

### Pediatric population and pharmacokinetic field study design.

In October 2012, 60 children from a wider ongoing study cohort were enrolled from two villages, Bugoigo and Walakuba, along the shores of Lake Albert, Uganda. They were initially selected on the basis of the history of *S. mansoni* infection as determined during previous surveys (36). All children had been previously treated with praziquantel (PZQ) (40 mg/kg of body weight) at least 1 year prior to the current study and had tolerated that treatment well. The study pediatrician (ALB) performed individual clinical assessments to establish suitability for participation in the study. The 60 subjects were randomized 1:1 to receive a single dose of 40 mg/kg or 60 mg/kg of PZQ. Exclusion criteria were as follows: (i) the child was acutely unwell as judged by the study pediatrician; and (ii) if the child was receiving other drug treatments, such as antimalarial agents that could potentially interact with PZQ. Side effects were recorded using a questionnaire before and 4 h after PZQ administration, and children were monitored for further symptoms for 24 h. These were classified as (i) self-reported and (ii) observed by the study pediatrician on site. Every child and parent in the study was also offered treatment for minor medical conditions. Figure 1 summarizes the sample design and basic methodology.

On the day of enrollment, each child arrived at the study site, which was a rural camp in Bugoigo village in Uganda. A breakfast consisting of local foods (doughnut, juice, and porridge) was provided before drug delivery, as food is known to increase PZQ bioavailability (4). PZQ at either 40 or 60 mg/kg was then administered. Venous blood samples (2 ml) were collected at time zero, 1 h, 2 h, 4 h, 6 h, 12 h, and 24 h into 5-ml heparinized tubes and centrifuged at 1,000 rpm for 10 min. Plasma was removed and immediately frozen in liquid nitrogen before being sent to the United Kingdom and The Netherlands on dry ice for further analysis. Children stayed on the premises for 24 h and were closely monitored for delayed drug-related adverse effects.

### Parasitological detection and pharmacodynamic endpoints.

Two stool samples (taken on consecutive days) and one urine sample were collected from every child prior to the start of the study. Double fecal smears were prepared from each sample applying the thick Kato-Katz technique for the detection of *S. mansoni* and other soil-transmitted helminths (37). Mean egg counts per gram of stool (epg) were subsequently calculated, and three categories were considered for *S. mansoni* infection: light (1 to 99 epg), medium (100 to 399 epg), and heavy (>400 epg) (6). Following WHO guidelines for the assessment of the drug efficacy of PZQ against schistosomiasis, standard measures of parasitological cure were used as primary study endpoints: egg reduction rate (ERR) (the percentage decrease in egg count between baseline and follow-up after treatment) and cure rate (CR) (the percentage of children with no schistosomal eggs at follow-up after treatment) with an acceptable CR of >85% set by the WHO (3).

Testing for circulating cathodic antigen (CCA) in urine was performed using the point-of-care CCA rapid diagnostic test (Rapid Medical Diagnostics, Pretoria, South Africa). The strips were graded upon visual inspection as trace, +, ++, and +++ (38). Frozen plasma samples from the subjects were later sent to the Leiden University Medical Center, The Netherlands, to be tested for the presence of circulating anodic antigen (CAA) levels by the up-converting phosphor lateral flow (UCP-LF)-based CAA assay (10). As the CAA levels in the plasma samples were very high, they were tested in 1/10 and 1/100 dilutions depending on the expected worm burden of the child. The cutoff for positivity of the assay was 10 pg/ml CAA plasma, corresponding to 10 worm pairs.

Rapid diagnostic tests were also used for the detection of malaria (First Response, Goa, India) and HIV (Determine; Alere, San Diego, CA, USA). A confirmatory test was available for any HIV tests that were initially positive (StandardDiagnostics HIV-1/2, Gyeonggi-do, Republic of Korea).

### Quantification and resolution of PZQ racemate and *R* and *S* enantiomers.

Plasma *R*-PZQ and *S*-PZQ concentrations were determined using a validated liquid chromatography-tandem mass spectrometry (LC/MS/MS) assay (39). Chromatographic separation was achieved using a gradient program. The ultrahigh-performance liquid chromatography (UHPLC) system was interfaced with a triple-quadruple TSQ Quantum Access mass spectrometer (Thermo Scientific, Hemel Hempstead, United Kingdom) with a heated electrospray ionization (H-ESI) source. An E2M28 rotary vacuum pump (Edwards High Vacuum International, West Sussex, United Kingdom), an NM30LA nitrogen generator (Peak Scientific, Renfrewshire, United Kingdom), and 99% pure argon gas (10 liters) (BIP10; Air Products, Liverpool, United Kingdom) were used. Plasma samples (500 µl) containing diazepam as an internal standard were extracted with a mixture of *methyl-tert*-butyl ether and dichloromethane (2:1, vol/vol), and the resulting dried extract was reconstituted in mobile phase and injected into the LC/MS/MS. The assay was linear in the range from 5 to 1,500 ng/ml for both PZQ isomers. The detailed methodology is described elsewhere (39).

### Pharmacokinetic population analyses.

A population methodology using the program Pmetrics (version 1.2.6) (University of Southern California) was used throughout (http://www.lapk.org). Two structural models were constructed and fitted to the data. In the first model, total PZQ concentrations (i.e., *R*-PZQ plus *S*-PZQ) were assessed. A standard three-compartment structural pharmacokinetic model was used. The three compartments were an absorptive compartment (i.e., gut), a central compartment (bloodstream), and a peripheral compartment. The parameters (units shown in brackets or parentheses) describing this model included a first-order rate constant connecting the gut with the bloodstream (*K_a_* [hour^−1^]), two first-order intercompartmental rate constants connecting the central and peripheral compartments (*K*_cp_ [hour^−1^] and *K*_pc_ [hour^−1^]), first-order clearance from the central compartment (SCL [l/hour]), volume of the central compartment (Vc [liters]), and an absorption lag (hours). Oral bioavailability was not estimated. To describe the population pharmacokinetics of the *R* and *S* isoforms, a separate structural model was developed (data not shown). Potential relationships between each model parameter and covariates (e.g., weight, age, and gender) were explored by plotting the Bayesian posterior estimate for the parameter against the covariate. Covariates that demonstrated a statistically significant relationship with the Bayesian parameter estimates were then incorporated into the structural model.

The fit of the model to the data was assessed by mean weighted error (a measure of precision), by mean weighted squared error (a measure of bias), and by visual inspection and coefficient of determination (*r*^2^) of the linear regression of the observed and predicted values both before and after the Bayesian step.

### Pharmacodynamics of PZQ.

All data were entered into electronic format using EpiData (The EpiData Association, Odense, Denmark) and analyzed using the R statistical package version 3.2.2. (The R Foundation for Statistical Computing, Vienna, Austria). Cure rate (CR) by egg clearance at 24 days was presented as percent egg negative by two Kato-Katz thick smears at follow-up with the associated exact binomial 95% confidence interval (95% CI). Multivariable logistic regression models were used to explore the relationship between CR and demographic and drug exposure parameters with unadjusted models and models adjusting for initial disease burden (egg count). A larger model also adjusting for child weight and sex was considered and gave very similar parameter estimates. Importantly, however, this model was overfitted given the relatively small number of events and is therefore not presented here. Since the baseline intensity prevalence was high, success was also analyzed as egg reduction rate (ERR) and not solely CR. The ERR was calculated as described elsewhere (3). A 95% CI and comparisons between dose groups used a quasibinomial regression model to allow for the overdispersion due to within-child correlations. Secondary outcomes included antigenic clearance by CCA and CAA levels, and these were explored descriptively.

### Monte Carlo simulation.

Monte Carlo simulations were performed using ADAPT 5 (40) and R. The first structural pharmacokinetic model using total PZQ concentrations was used for the purposes of computational tractability and to enable future correlation with studies where *R* and *S* enantiomers are not measured. Because of persistent bias when using the population parameter estimates (with both mean or median parameter values), we chose not to use these values and the associated population covariance matrix for the Monte Carlo simulations. Rather, we used the Bayesian posterior values for each patient, because the individual predicted values versus observed values were unbiased (Fig. 3). We generated 100 simulated patients from each patient’s posterior estimates, using their mean parameter estimates and the individual’s covariance matrix. Three regimens were considered in these simulations: 40 mg/kg, 60 mg/kg, and 80 mg/kg of PZQ. The AUC was computed for each simulated patient using integration.

A cure model based on a logistic regression fit to the cure rate that included baseline egg number and total AUC was used to estimate the cure probability for each patient. As there was only limited outcome data, it was not possible to reliably model covariates, such as weight and sex. The baseline egg count was sampled from the distribution observed in the treated patients. In order to estimate the errors in the simulated estimates, 1,000 sets of logistic regression parameters were simulated based on draws from the multivariate normal distribution of the parameter estimates and their covariance matrix in the logistic regression model. The median and interquartile ranges of the cure rates for the simulated populations are presented. Additionally, the proportion of simulated patients achieving a predicted cure rate of >85% was computed.

## SUPPLEMENTAL MATERIAL

Figure S1  Adverse events that were self-reported (A) or observed by pediatrician (B). Download Figure S1, JPG file, 0.4 MB
