# Supplementary figures and images for: Population Pharmacokinetics and Pharmacodynamics of Praziquantel in Ugandan Children with Intestinal Schistosomiasis: Higher Dosages Are Required for Maximal Efficacy
Source: mBio. 2016 Aug 9;7(4):e00227-16. doi: 10.1128/mBio.00227-16 (PMC4992966; doi:10.1128/mBio.00227-16)

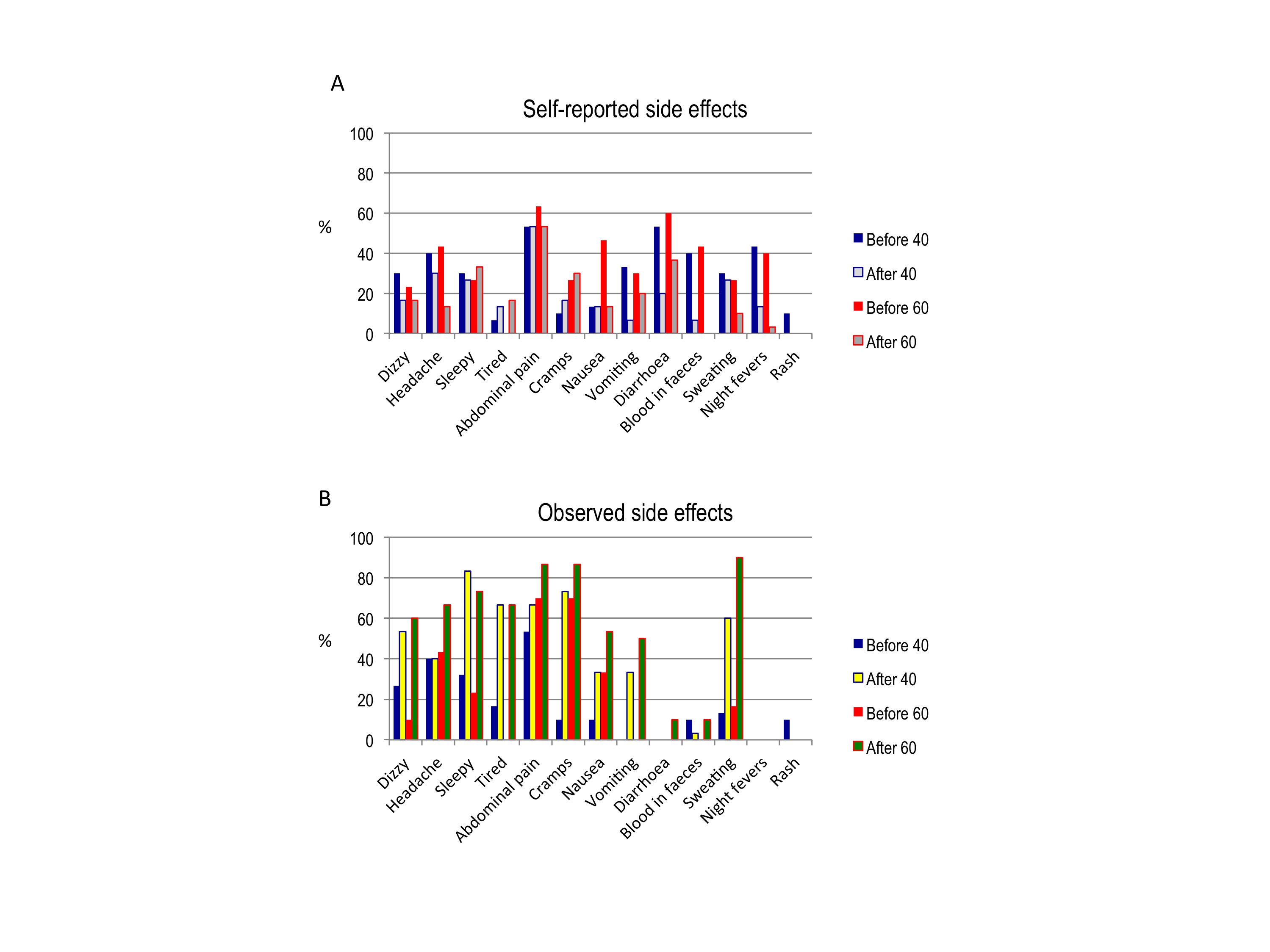

Supplement: Figure S1 — Adverse events that were self-reported (A) or observed by pediatrician (B). Download [file mbo004162930sf01.jpg]
